# Effects of an Internet of Things-Based Medication Assistance System on Real-World ART Adherence and Treatment Response in People Living with HIV

**DOI:** 10.3390/jcm15031151

**Published:** 2026-02-02

**Authors:** Jin Woong Suh, Kyung Sook Yang, Jeong Yeon Kim, Young Kyung Yoon, Jang Wook Sohn

**Affiliations:** 1 Division of Infectious Diseases, Department of Internal Medicine, Kyung Hee University College of Medicine, Kyung Hee University Hospital at Gangdong, Seoul 05278, Republic of Korea; sunthes@naver.com; 2 Department of Biostatistics, Korea University College of Medicine, Seoul 02841, Republic of Korea; 3 Division of Infectious Diseases, Department of Internal Medicine, Korea University College of Medicine, Seoul 02841, Republic of Korea; 4 Institute of Emerging Infectious Diseases, Korea University, Seoul 02841, Republic of Korea

**Keywords:** HIV, antiretroviral therapy, internet of things, adherence

## Abstract

**Background/Objectives**: The study primarily examined whether an IoT-based medication assistance system enhances ART adherence relative to standard care, and secondarily evaluated device feasibility and error patterns over time. **Methods**: This prospective study was conducted between June 2022 and October 2023 at a tertiary hospital in South Korea. Adults (≥19 years) living with HIV and prescribed ART were included; those with comorbid hepatitis B or C were excluded. People living with HIV who agreed to use the IoT-based InPHRPILL system (Sofnet Inc., Seoul, Republic of Korea) were assigned to the intervention group, whereas those who declined were assigned to the control group. Viral suppression, CD4^+^ cell counts, and adherence rates were measured. Additional analyses evaluated 12-month longitudinal adherence using pill-count data in both groups, and device-measured adherence and device-associated error rates in the intervention group. **Results**: Thirty-five participants (12 in the intervention group and 23 in the control group) were included. The intervention group demonstrated marginally shorter durations since HIV diagnosis and ART initiation at study enrollment, as well as slightly higher baseline HIV-RNA levels; however, these differences did not reach statistical significance. The median pill-counting and IoT device adherence rates were 100% and 87.4%, respectively (median deviation error rate = 4.4%). Poisson regression revealed significantly reduced error rates over time (*β* = −0.06493, *p* < 0.01), suggesting improved device use proficiency. **Conclusions**: IoT-based medication assistance systems may provide objective, real-time monitoring of ART adherence and facilitate identification of discrepancies between clinical evaluations and actual adherence patterns. Larger studies targeting individuals with suboptimal adherence are warranted to determine whether such systems can enhance adherence outcomes.

## 1. Introduction

Globally, human immunodeficiency virus (HIV) infections remain a critical public health crisis affecting 39.9 million people and causing 630,000 deaths by 2023 [1]. Over the past few decades, significant advances in antiretroviral therapy (ART) have improved the outcomes of people living with HIV. For example, ART extends life expectancy by 37 years in people living with HIV who start treatment at 20 years of age [2]. In line with these improvements, the United Nations Programme on HIV/AIDS (UNAIDS) and the WHO have set ambitious “95–95–95” target to end the HIV/AIDS epidemic by 2030 by focusing on achieving viral suppression in 95% of people receiving ART. However, achieving the third “95”—viral suppression—depends on high ART adherence rates, as even minor lapses can result in viral rebound and resistance as well as treatment failure [3]. Current research emphasizes that adherence levels ≥ 80% are essential, with US Centers for Disease Control findings suggesting that an 82% adherence rate achieves viral suppression in 90% of cases, while specific regimens achieve suppression with adherence rates as low as 75% [4]. Despite this, global adherence rates often fall short of the optimal 95% with averages of 74.1% and 70.4% in the United States and South Korea, respectively [3,5]. Therefore, improving adherence to ART has become a central focus in research of people living with HIV.

Recent studies have highlighted the potential of digital tools such as SMS reminders, mobile health applications, and electronic monitoring to enhance ART adherence rates, particularly in resource-limited settings [6,7]. In addition, Internet of Things (IoT)-based technologies such as smart pill bottles and wearable devices offer real-time tracking, reminders, and data sharing to improve adherence management [8,9]. Previous meta-analyses have reported that IoT-based technologies, particularly telehealth interventions, show an 18% higher adherence rate in telehealth-supported versus control groups [10]. The integration of these IoT tools into the care of people living with HIV can further enhance treatment adherence strategies and provide continuous support outside the clinical setting [11,12]. However, further research is required to assess the efficacy of these interventions comprehensively. Accordingly, this investigation primarily examined whether an IoT-based medication assistance system (InPHRPILL; Sofnet Inc., Seoul, Republic of Korea) enhances ART adherence relative to standard care among people living with HIV in a real-world clinical environment and secondarily assessed the feasibility of device-based monitoring and associated error patterns over time.

## 2. Materials and Methods

### 2.1. Study Population

**Figure 1:**
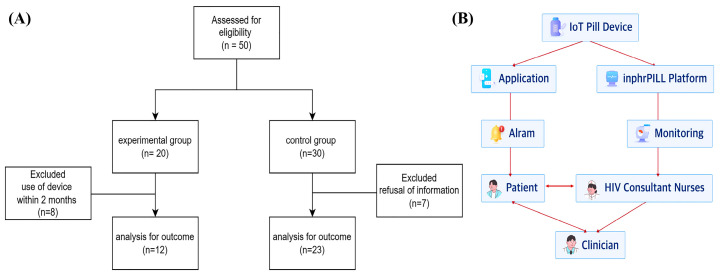
Patient enrollment process (**A**) and relationships among IoT device users, consultant nurses, and clinicians (**B**) during the study period.

This single-center prospective study was conducted between June 2022 and October 2023 at a tertiary hospital in the Republic of Korea. Adult people living with HIV (≥19 years) were prescribed bictegravir/emtricitabine/tenofovir alafenamide (Biktarvy; Gilead Sciences Inc., Mississauga, ON, Canada), dolutegravir/lamivudine (Dovato; ViiV Healthcare, London, UK), dolutegravir/abacavir/lamivudine (Triumeq; ViiV Healthcare), dolutegravir (Tivicay; ViiV Healthcare). Additionally, emtricitabine/tenofovir alafenamide (Descovy; Gilead Sciences Inc.) was used as part of combination therapy. Individuals with HIV and comorbid hepatitis B or C were excluded from the study. People living with HIV were assigned to the intervention group if they voluntarily agreed to use the IoT-based medication assistance system and to the control group if they declined its use; thus, group allocation was not randomized. Participants were prospectively recruited between June 2022 and October 2023 at Korea University Anam Hospital, Seoul, Republic of Korea. The study groups are shown in Figure 1A.

### 2.2. Follow-Up Assessments

Throughout the study period, all people living with HIV continued their existing treatment regimens and underwent routine laboratory tests at baseline, pre-intervention, and post-intervention (6 and 12 months) as per standard clinical practice [13]. Follow-ups were conducted during clinic visits, during which HIV counseling nurses collected adherence data for the intervention and control groups using pill counts.

### 2.3. Adherence and Error Rates

ART adherence rates based on pill counts were analyzed during routine clinical visits. The pill-counting adherence rate was calculated as the percentage of pills taken out of the total prescribed during routine clinical visits by HIV counseling nurses. The device-associated adherence rate was defined as the percentage of days with recorded medication intake out of the total number of days in a month according to the InPHRPILL system. To evaluate the errors associated with the monitoring devices, the device-associated error rate was defined as the percentage of days with two or more instances of recorded medication intake out of the total number of days per month.

### 2.4. IoT-Based Medication Assistance System Usage Monitoring

The intervention group agreed to use the InPHRPILL system (Sofnet Inc.), along with IoT-based medication assistance devices and apps (Coledy Inc., Seoul, Republic of Korea). The IoT-based medication assistance devices consisted of pill dispensers equipped with sensors located on their lids to detect when the medication is dispensed. These devices operate wirelessly in conjunction with an app that enables users to set medication schedules and receive reminders Together, the InPHRPILL system and IoT-enabled app form an integrated platform that enables real-time adherence monitoring.

The IoT device recorded medication intake events and provided immediate feedback to users through the app while simultaneously transmitting adherence data to HIV counseling nurses via the InPHRPILL system. Adherence data from the IoT-based devices were validated by cross-referencing with the pill counts recorded during clinic visits. HIV counseling nurses mediated adherence data between clinicians and patients using this system. This dual feedback mechanism was designed to enhance adherence management by promoting user engagement and enabling timely clinical interventions (Figure 1B). A visual overview of the smart pill dispenser and the InPHRPILL login interface is presented in Supplementary Figure S1.

### 2.5. Definitions and Outcomes

Baseline characteristics and clinical variables were extracted from electronic medical records, including age, sex, duration since HIV diagnosis, interval from ART initiation to study enrollment, HIV-RNA levels and CD4^+^ cell counts at baseline, pre-intervention, and follow-up visits. Baseline HIV-RNA was defined as the measurement obtained at the time of HIV diagnosis, whereas pre-intervention HIV-RNA referred to the most recent measurement prior to study enrollment. Viral suppression was defined as HIV-RNA <20 copies/mL. At enrollment, all participants were receiving ART. The ART duration at study enrollment was calculated from the date of the first ART prescription to the date of enrollment. The primary outcome was the between-group difference in ART adherence during follow-up, assessed using pill-count–based adherence collected at routine clinic visits. The secondary outcomes (intervention group only) included device-measured adherence, device-associated error rates, and their longitudinal changes over time as indicators of user adaptation and proficiency. Exploratory outcomes were HIV-RNA suppression and CD4^+^ cell counts during follow-up. The error rate was defined as the percentage of days during the entire follow-up period in which the medication was recorded as being dispensed more than twice per day.

### 2.6. Subgroup Analysis of Adherence Rates of Pill-Counting Versus Medication Assistance Device in the Intervention Group

A subgroup analysis was performed on the intervention group to assess the monthly ART adherence and error rates of the IoT-based medication assistance system over a 12-month period. The ART adherence rates in the subgroup analysis were evaluated using pill counting and device-measured adherence rates. During follow-up clinic visits, adherence data were further supplemented by device-associated adherence reports and pill-counting data.

### 2.7. Statistical Analysis

The priori power analysis conducted with G*Power (ver. 3.1.9.4) indicated that 64 participants per group (128 total) were required to detect a medium effect size (Cohen’s *d* = 0.5) with 80% power at a two-tailed α of 0.05 using an independent samples *t*-test. However, due to recruitment constraints, only 12 participants were enrolled in the intervention group and 23 in the control group. Under the original assumptions, this sample size yielded an achieved power of approximately 26.6%. Accordingly, the study was underpowered to detect modest between-group differences, and the findings should be regarded as exploratory.

Categorical variables are expressed as counts or frequencies and were analyzed using the chi-squared or Fisher’s exact test, while continuous variables were analyzed using the Student’s *t*-test or Mann–Whitney U test. Baseline characteristics are summarized as frequencies (percentages) for categorical variables and medians with interquartile ranges (IQR) for continuous variables. Multivariate logistic regression was conducted to explore factors associated with membership in the perfect-adherence group. The results are expressed as odds ratios (OR) with 95% confidence intervals (CIs). Statistical significance was set at *p* < 0.05.

Longitudinal changes in device-associated error counts were analyzed using a Poisson mixed-effects framework with subject-specific random intercepts. This approach accounts for correlation among repeated monthly measurements within individuals and accommodates between-person heterogeneity in baseline error propensity. Neglecting within-subject correlation may result in underestimated standard errors, and overly narrow confidence intervals. Duration (months since device initiation) was included as a fixed effect. Additionally, overdispersion was assessed, and comparable findings were obtained using a negative binomial model. The model estimates the log of the expected error count as a linear function of time while allowing each participant to have their own baseline error rate. The results are presented as regression coefficients (*β*) and incidence rate ratios (IRR) with corresponding 95% CIs. Statistical significance was defined as a two-sided *p*-value of <0.05. Within the intervention group, longitudinal associations between follow-up duration and device-associated adherence/error measures were examined using the mixed-effects count model described previously.

## 3. Results

### 3.1. Baseline Characteristics of People Living with HIV

The primary objective of this study was to assess whether the IoT-based system enhanced ART adherence compared with controls, while secondary analyses examined device-measured adherence and error patterns within the intervention group. Of the 50 people living with HIV, 35 were enrolled (12 in the intervention group using the IoT-based medication assistance system and 23 in the control group) (Figure 1A). As shown in Table 1, the median age of the cohort was 36 years (IQR: 27–49 years), and all participants were male. The control group included a higher proportion of participants aged >35 years relative to the intervention group (*p* = 0.04). Although these differences between-group were not statistically significant, the intervention group had slightly shorter times since HIV diagnosis and ART initiation at study enrollment, as well as marginally higher baseline HIV-RNA levels at diagnosis. Viral suppression, defined as HIV-RNA < 20 copies/mL, increased from 6/35 (17.1%) at diagnosis to 25/35 (71.4%) at the pre-intervention time point, with lower suppression observed in the intervention group than in the control group (41.7% vs. 87.0%, *p* = 0.005). Suppression further increased to 31/35 (88.6%) at 6 months and reached 35/35 (100%) at 12 months following the intervention. Table 2 presents an exploratory analysis of factors associated with maintaining perfect pill-count–based ART adherence during follow-up. The two groups in Table 2 were defined as follows: Participants whose pill-count–based adherence remained at 100% throughout follow-up were classified as the perfect-adherence group, whereas those with pill-count–based adherence <100% at any follow-up visit were classified as the imperfect-adherence group. This pill-count based classification was used for exploratory purposes and was not a prespecified primary outcome. Univariate analysis did not identify any statistically significant factors influencing adherence rates. The multivariate logistic regression analysis did not identify any statistically significant predictors of ART adherence (Table 3).

### 3.2. Subgroup Analysis of Pill-Counting Versus Device-Measured Adherence and Error Rates in Intervention Group

The baseline characteristics of the intervention group (*n* = 12; all males; median age, 30 years) are summarized in Table 4. The median duration of device use was 10.5 months, reflecting consistent engagement with the IoT-based medication assistance system throughout the study period. This study revealed a significant difference between the pill-counting and device-measured adherence rates. The median pill-counting adherence rate was exceptionally high at 100%, suggesting that the patients believed they were closely following their prescribed medication regimens. However, the IoT was lower (87.4%). This discrepancy highlights the gap between the subjective people living with HIV adherence and the objective data obtained from the device. The ability of the device to provide real-time, unbiased measurements offers valuable insights into actual medication-taking behaviors, which may differ from the self-reports of people living with HIV. The median error rate, defined as the percentage of days per month with two or more instances of medication being dispensed from the device, was 4.4%. This error rate suggests potential deviations from the prescribed medication schedule, possibly due to forgetfulness or other factors.

### 3.3. Subgroup Analysis of the Device Error Rates of the Intervention Group

A Poisson regression model with random intercepts was used to analyze the error rates associated with device usage over time (Table S1). The coefficients for the monthly error rates were statistically significant (*β* = −0.06493, *p* < 0.01), indicating a continuous reduction in error rates over time. This trend suggests that people living with HIV become more proficient in using the device over time, leading to fewer errors in medication intake. Interestingly, the analysis also revealed negative effects during specific months when error rates had a more pronounced impact on the outcome. This observation implies that external factors such as lifestyle changes or psychological barriers may have influenced patient adherence rates during the study period. These fluctuations in error rates highlight the complexity of maintaining consistent adherence to ART and the challenges that people living with HIV face when integrating medication-taking routines into their daily lives.

## 4. Discussion

This study evaluated the real-world application of an IoT-based medication assistance system for monitoring ART adherence among people living with HIV. Although no statistically significant difference in adherence was observed between the IoT intervention group and the control group, the findings provide valuable insights into adherence assessment and monitoring within contemporary HIV care. Previous studies have demonstrated the application of IoT technology in the management of various diseases. For example, one study reported that an IoT-based smart medication management system helped people living with HIV effectively manage their hypertension [14], whereas another study indicated a substantial increase in adherence and completion rates among people living with HIV and tuberculosis using smart medication dispensers [15,16]. The findings suggest that similar IoT-based systems can enhance the precision of adherence monitoring, enabling clinicians to identify adherence patterns and implement timely, targeted interventions tailored to individual patient needs.

The absence of a significant between-group difference should be interpreted in relation to the study population’s baseline adherence profile. At study entry, most participants exhibited very high adherence to ART, with pill-count–based adherence rates approaching 100%. In populations with pre-existing high ART adherence, a ceiling effect may constrain the potential of adherence-enhancing interventions to yield measurable improvements. Consequently, IoT-based systems in such settings may function primarily as objective monitoring tools rather than as interventions designed to further elevate adherence rates. Despite this limitation, the clinical utility of objective, real-time adherence monitoring remains substantial. Precise identification of adherence lapses, even when overall adherence is high, facilitates early detection of treatment failure risk and supports proactive clinical management [17,18]. Furthermore, the capacity to differentiate between truly adherent patients and those who inaccurately report adherence provides critical information for treatment optimization and clinical decision-making. As baseline HIV-RNA was defined at the time of HIV diagnosis, viral suppression was predictably low at baseline and higher at the pre-intervention assessment, reflecting ART exposure prior to study enrollment. Notably, viral suppression at the pre-intervention time point varied between groups, potentially indicating baseline imbalance arising from the non-randomized allocation, which should be considered when interpreting between-group comparisons.

In our study, the mean age of the participants in the intervention group was 30 years, representing a predominantly young adult population. Although adherence assessed in the clinic setting was 100%, the actual adherence measured by the IoT device was 87.4%. This discrepancy underscores a critical gap between perceived and actual adherence patterns, underscoring the limitations of conventional clinic-based adherence assessment methods such as self-report and pill-count approaches. Young adults living with HIV face significant challenges in maintaining consistent ART adherence, making this group a critical target for intervention [19]. For instance, studies have shown that more than one-third of adolescents and young adults living with HIV exhibit suboptimal adherence owing to diverse social, psychological, and structural barriers [20]. However, effective interventions require precise detection of adherence lapses and the timing of their occurrence. A pilot randomized trial demonstrated that mobile apps use improved self-reported ART adherence during active intervention, though these benefits diminished when app access was withdrawn, highlighting the challenges in sustaining long-term engagement and behavioral change with digital health interventions [12]. Unlike clinic-based measures, continuous IoT monitoring can capture the timing and patterns of missed doses more objectively. This may allow clinicians to provide timely, targeted support rather than relying on generic adherence counseling. Taken together, our findings support the feasibility of IoT-based adherence monitoring and suggest its potential utility in young adults who face substantial adherence barriers.

The median device-measured adherence rate in the intervention group (87.4%) exceeded the real-world average previously reported in the United States (74.1%) between 2017 and 2019 [3]. This suggests that ART adherence rates are not consistently high in real-world settings and highlights the challenges of maintaining them at >90% among people living with HIV, similar to findings from real-world data in the United States [3]. However, despite an average adherence rate of <90%, our study showed that viral suppression was maintained. Previous studies have linked low ART adherence rates with a higher risk of virologic failure, which refers to the inability to achieve or maintain suppression of viral replication below the detectable level. For example, one study found that people living with HIV with an adherence rate of <80% had double the risk of virologic failure [21]. Another study indicated that suboptimal adherence could lead to detectable viremia, decreased CD4^+^ counts, viral resistance, increased transmission, earlier death, and poor overall quality of life [22]. However, recent studies have shown that people living with HIV who maintain a proportion of days covered (PDC) adherence rates ≥75% achieve viral suppression success rates comparable to those with adherence rates >90% [23]. This suggests that while high adherence remains crucial for viral suppression, the efficacy of current ART regimens may reduce the need for traditionally targeted adherence rates >90%. However, achieving adherence is often challenging due to social factors such as the stigma of taking medication in public, medication side effects, concurrent mental health conditions, alcohol and/or drug use, perceptions of drug efficacy, and medication regimen complexity [9,24].

The longitudinal analysis revealed a significant decline in device-associated error rates over time, reflecting improved user familiarity and adaptation to the IoT system. This learning effect is clinically relevant, as prior research has demonstrated that technical issues and user errors in digital adherence systems can compromise treatment outcomes by disrupting medication routines and diminishing patient trust in technology [9,25,26]. Although the error rates decreased over time in the current study, thereby suggesting improved user familiarity with the device, periods of elevated errors may reflect challenges such as technical glitches or difficulties in system use. Addressing these issues through device design enhancement, user education, and proactive technical support can reduce errors and improve the adherence rate of people living with HIV. Future research should explore the link between error rates and adherence to ensure that IoT-based systems are both reliable and user-friendly in order to maximize their clinical impact.

The strength of this study lies in the innovative application of IoT-based smart medication management systems to improve adherence rates among people living with HIV. This dual feedback mechanism may facilitate more effective adherence monitoring and enable timely, targeted support by providing real-time feedback to users and adherence reports to clinicians. This approach offers a tailored strategy for integrating objective adherence data into routine HIV care.

This study has several limitations. First, the relatively small sample size limited the statistical power to detect modest differences between the intervention and control groups. In addition, participants exhibited relatively high baseline ART adherence, resulting in a ceiling effect that constrained the potential for further measurable improvement. Consequently, the lack of a statistically significant effect of the IoT-based intervention should be interpreted as a characteristic of the study population rather than as evidence of the intervention’s ineffectiveness. Second, participants were not randomized to the intervention or control groups, as allocation was based on willingness to use the IoT system. This non-randomized design may have introduced selection bias and baseline imbalances, although multivariable analyses were performed to adjust for key confounders. Third, the study was conducted at a single center, which may restrict the generalizability of the findings to other clinical settings or populations. Furthermore, the follow-up duration may not have been sufficient to fully capture long-term adherence patterns and clinical outcomes associated with IoT-based monitoring. Despite these limitations, the study provides valuable real-world evidence regarding the feasibility of IoT-enabled adherence monitoring and underscores important considerations for future research, particularly the need to focus on populations with greater potential to benefit from digital adherence interventions.

## 5. Conclusions

This study suggests that IoT-based systems can deliver objective, real-time adherence monitoring, that may reveal discrepancies between conventional clinic-based assessments and actual adherence behavior. To maximize clinical benefit, future research should prioritize populations with documented adherence challenges and design scalable implementation models for routine clinical integration.

## Figures and Tables

**Table 1:** Baseline characteristics of patients with HIV using IoT-based medication assistance system.

Characteristic	Total	Intervention Group (*n* = 12)	Control Group (*n* = 23)	*p*-Value
Male sex, *n* (%)	35 (100)	12 (100)	23 (100)	
Age (years), median (IQR)	36 (27–49)	30 (26–45)	37 (30–55)	0.10
Age, >35 years, *n* (%)	20 (57.1)	4 (33.3)	16 (69.6)	0.04
Duration since HIV diagnosis (months), median (IQR)	43.6 (15.7–81.5)	20.4 (4.7–70.1)	49 (28.4–90.1)	0.49
ART duration at study enrollment(months), median (IQR)	40.1 (15.8–81.2)	25.7 (5.1–65.5)	50.4 (26.4–87.3)	0.48
Adherence (%), median (IQR)				
Pre pill-counting	100 (98.6–100)	100 (98.7–100)	100 (97.7–100)	0.45
Post pill-counting	100 (98.8–100)	100 (97.2–100)	100 (98.8–100)	0.26
Pill-counting adherence increase, *n* (%)	22 (62.9)	9 (75.0)	13 (56.5)	0.28
Baseline HIV-RNA (copies/mL), median (IQR)	56,100 (739–205,000)	63,750 (97–552,500)	56,100 (3260–104,000)	0.88
Viral suppression *, *n* (%)				
Baseline	6 (17.1)	3 (25.0)	3 (13.0)	0.37
Pre-intervention	25 (71.4)	5 (41.7)	20 (87.0)	0.005
Post-intervention (6 months)	31 (88.6)	9 (75.0)	22 (95.7)	0.068
Post-intervention (12 months)	35 (100)	12 (100)	23 (100)	-
CD4^+^ cell count (/μL), median (IQR)				
Baseline	356 (149–519)	379 (141–768)	356 (149–489)	0.20
Pre-intervention	527 (356–828)	552 (337–665)	513 (356–829)	0.76
Post-intervention (6 months)	544 (361–782)	529 (305–725)	545 (361–837)	0.78
Post-intervention (12 months)	491 (386–792)	717 (428–971)	481 (377–762)	0.18

* Viral suppression defined HIV-RNA < 20 copies/mL. IQR, interquartile range; HIV, human immunodeficiency virus; ART, antiretroviral therapy; RNA, ribonucleic acid.

**Table 2:** Exploratory analysis of factors associated with changes in pill-count based ART adherence.

Variable	Total (*n* = 35)	Imperfect-Adherence Group (*n* = 13)	Perfect-Adherence Group (*n* = 22)	*p*-Value
Intervention group	12 (34.3)	3 (23.1)	9 (40.9)	0.28
Male sex, *n* (%)	35 (100)	13 (100)	22 (100)	
Age (years), median (IQR)	36 (27–49)	34 (26–41)	42 (30–55)	0.25
Age > 35 years, *n* (%)	20 (57.1)	6 (46.2)	14 (63.6)	0.31
Duration since HIV diagnosis (months), median (IQR)	43.6 (15.7–81.5)	33.4 (13.2–75.4)	49.1 (15.8–93.1)	0.52
ART duration at study enrollment (months), median (IQR)	40.1 (15.8–81.2)	33.3 (12.7–74.7)	64.2 (17.5–92.4)	0.49
Baseline HIV-RNA (copies/mL), median (IQR)	56,100 (739–205,000)	104,000 (6994–1,019,000)	32,650 (559–101,000)	0.22
Viral suppression *, *n* (%)				
Baseline	6 (17.1)	1 (7.7)	5 (22.7)	0.254
Pre-intervention	25 (71.4)	11 (84.6)	14 (63.6)	0.184
Post-intervention (6 months)	31 (88.6)	10 (76.9)	21 (95.5)	0.096
Post-intervention (12 months)	35 (100)	13 (100)	22 (100)	-
CD4^+^ cell count (/μL), median (IQR)				
Baseline	356 (149–519)	476 (135–544)	356 (196–486)	0.58
Pre-intervention	527 (356–828)	527 (299–675)	545 (378–852)	0.36
Post-intervention (6 months)	544 (361–782)	477 (293–607)	562 (394–803)	0.36
Post-intervention (12 months)	491 (386–793)	481 (381–730)	533 (385–913)	0.37

* Viral suppression defined HIV-RNA < 20 copies/mL. IQR, interquartile range; HIV, human immunodeficiency virus; ART, antiretroviral therapy; RNA, ribonucleic acid.

**Table 3:** Logistic regression analysis of factors influencing perfect ART adherence among patients with HIV.

Variables	*β*-Coefficient	Standard Error	Odds Ratio	95% CI	*p*-Value
IoT device use	1.229	0.867	3.416	0.625–18.680	0.16
Age > 35 years	1.190	0.790	3.288	0.699–15.461	0.13

CI, confidence interval; IoT, internet of things; ART, antiretroviral therapy; HIV, human immunodeficiency virus.

**Table 4:** Subgroup analysis of pill-counting versus device-measured adherence rates in intervention group of patients with HIV.

Variable	Intervention Group
Age (years), median (IQR)	30 (26–45)
Months of HIV infection, median (IQR)	20.4 (4.65–70.1)
Months of ART, median (IQR)	25.7 (5.08–65.48)
Duration of device use (months), median (IQR)	10.5 (8.25–16.5)
Adherence rate (%), median (IQR)	
Pre pill-counting	100 (98.68–100)
Post pill-counting	100 (97.18–100)
IoT device-counting	87.4 (78.75–92.18)
IoT device error rate (%), median (IQR)	4.4 (1.73–7.33)

HIV, human immunodeficiency; IQR, interquartile range; ART, antiretroviral therapy; IoT, internet of things.

## Data Availability

Owing to the sensitive nature of HIV-related clinical data and Institutional Review Board restrictions, participant-level data are not publicly accessible. De-identified data (where permitted) and analysis code may be obtained from the corresponding author upon reasonable request, subject to required approvals and data-use agreements.

## Supplementary Materials

The following supporting information can be downloaded at: https://www.mdpi.com/article/10.3390/jcm15031151/s1, Table S1: Adherence and errors of the IoT-based medication assistance system for ART in the intervention group of patients with HIV; Figure S1: Structural components of the IoT-based medication assistance system and its integrated user interface.

## Abbreviations

The following abbreviations are used in this manuscript:

HIV	Human immunodeficiency virus
ART	Antiretroviral therapy
UNAIDS	Joint United Nations Programme on HIV/AIDS
WHO	World Health Organization
IoT	Internet of Things
HIV-RNA	Human immunodeficiency virus ribonucleic acid
CD4	Cluster of differentiation 4 (T-lymphocyte subset)
IQR	Interquartile range
OR	Odds ratio
CI	Confidence interval
RCT	Randomized controlled trial
mHealth	Mobile health
IRR	Incidence rate ratio
