# Supplementary material for: Effects of an Internet of Things-Based Medication Assistance System on Real-World ART Adherence and Treatment Response in People Living with HIV
Source: J Clin Med. 2026 Feb 2;15(3):1151. doi: 10.3390/jcm15031151 (PMC12897846; doi:10.3390/jcm15031151)
Supplement: Supplementary file 1 [file jcm-15-01151-s001.zip › jcm-4034131-supplementary.pdf]

Journal of
Clinical Medicine

MDPI

Effects of an Internet of Things–Based Medication Assistance System on Real-World ART Adherence and Treatment Response in People living with HIV

Supplementary Material

Table S1. Adherence and errors of the IoT-based medication assistance system for ART in the intervention group of patients with HIV.

Effect	Estimate (*β*)	Standard Error	DF	T-value	*P*-value
**Adherence rate**					
**Intercept**	1.7273	0.1084	10	15.93	<0.01
**Age**	0.003249	0.002816	128	1.15	0.25
**Months**	- 0.00638	0.003836	128	-1.66	0.10
**Error rate**					
**Intercept**	- 0.5501	0.7658	10	-0.72	0.49
**Age**	- 0.00934	0.02120	128	-0.44	0.66
**Months**	- 0.06493	0.02127	128	-3.05	<0.01

ART, antiretroviral therapy; DF, degrees of freedom; HIV, human immunodeficiency virus; IoT, Internet of Things

Figure S1. Structural components of the IoT-based medication assistance system and its integrated user interface.

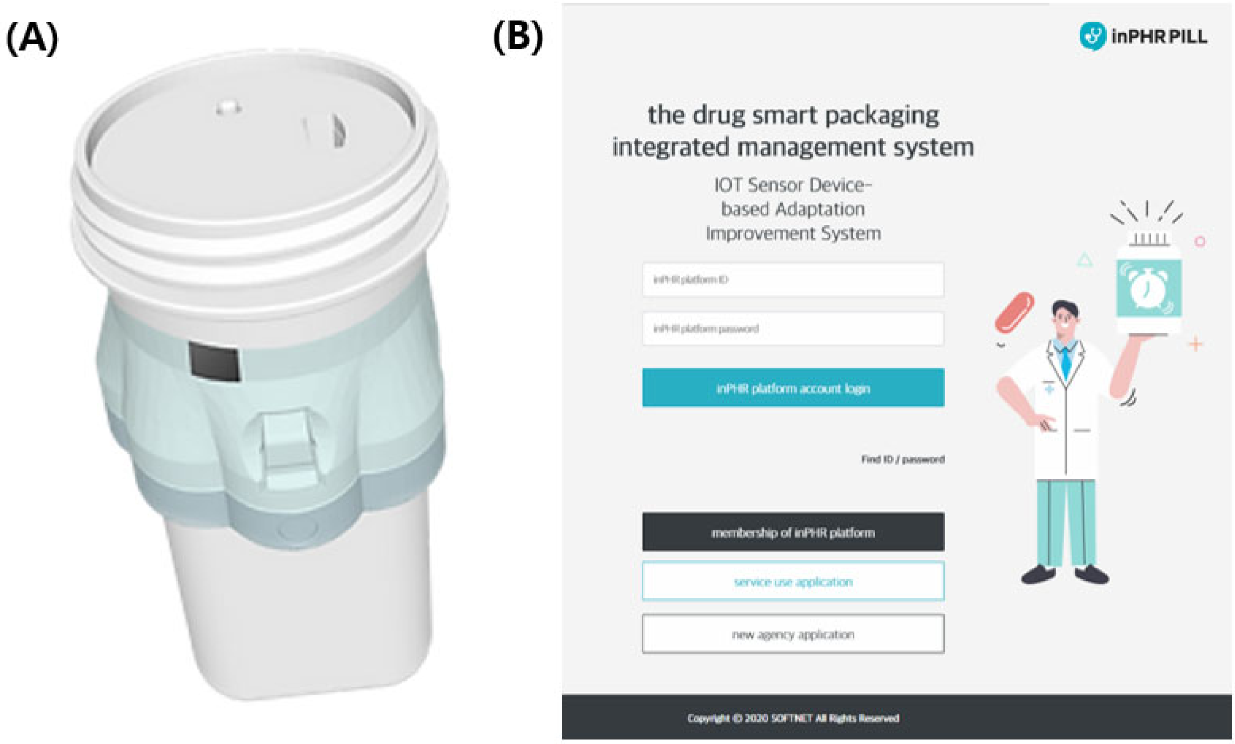

The device and platform were used together to support real-time medication dispensing/monitoring and to generate adherence-related records during the study period. (A) Photograph of the IoT-enabled smart pill dispenser used in this study, (B) Screenshot of the InPHRPILL login page used by clinicians to access the system.
